# Inhalative steroids as an individual treatment in symptomatic lung cancer patients with radiation pneumonitis grade II after radiotherapy – a single-centre experience

**DOI:** 10.1186/s13014-016-0580-3

**Published:** 2016-02-02

**Authors:** C. Henkenberens, S. Janssen, M. Lavae-Mokhtari, K. Leni, A. Meyer, H. Christiansen, M. Bremer, N. Dickgreber

**Affiliations:** Department of Radiation Oncology, Hannover Medical School, Carl-Neuberg-Str. 1, 30625 Hannover, Germany; Hannover Joint Practice in Radiooncology, Rundestr. 10, 30161 Hannover, Germany; Ibbenbüren Hospital Thoracic and Lung Center, Große Str. 41, 49477 Ibbenbüren, Germany; Hildesheim Goslar Joint Practice in Radiooncology, Senator-Braun-Allee, 31135 Hildesheim, Germany; Department of Pneumology, Thoracic Oncology and Respiratory Medicine, Frankenburgstr, 31, 48431 Rheine, Germany; Department of Radiation Oncology, University of Lübeck, Lübeck, Germany; Department of Radiotherapy and Special Oncology, Hannover Medical School, Carl-Neuberg-Str. 1, 30625 Hannover, Germany

**Keywords:** Radiation pneumonitis, Lung cancer, Inhalative steroids

## Abstract

**Purpose:**

To assess efficacy of our single-centre experience with inhalative steroids (IS) in lung cancer patients with symptomatic radiation pneumonitis (RP) grade II.

**Material and methods:**

Between 05/09 and 07/10, 24 patients (female, *n* = 8; male, *n* = 16) with lung cancer (non-small cell lung carcinoma [NSCLC]: *n* = 19; small cell lung cancer [SCLC]: *n* = 3; unknown histology: *n* = 2) and good performance status (ECOG ≤1) received definitive radiotherapy to the primary tumour site and involved lymph nodes with concurrent chemotherapy (*n* = 18), sequential chemotherapy (*n* = 2) or radiation only (*n* = 4) and developed symptomatic RP grade II during follow-up. No patient presented with oxygen requiring RP grade III. The mean age at diagnosis was 66 years (range: 50–82 years). Nine patients suffered from chronic obstructive pulmonary disease (COPD) before treatment, and 18 patients had a smoking history (median pack years: 48). The mean lung dose was 15.5 Gy (range: 3.0–23.1 Gy). All patients were treated with IS. If a patient’s clinical symptoms did not significantly improve within two weeks of IS therapy initiation, their treatment was switched to oral prednisolone.

**Results:**

All 24 patients were initially treated with a high dose IS (budesonide 800 μg 1-0-1) for 14 days. Of the patients, 18 showed a significant improvement of clinical symptoms and 6 patients did not show significant improvement of clinical symptoms and were classified as non-responders to IS. Their treatment was switched to oral steroids after two weeks (starting with oral prednisolone, 0.5 mg/kg bodyweight; at least 50 mg per day). All of these patients responded to the prednisolone. None of non-responders presented with increased symptoms of RP and required oxygen and / or hospitalization (RP grade III). The median follow-up after IS treatment initiation was 18 months (range: 4–66 months). The median duration of IS treatment and prednisolone treatment was 8.2 months (range: 3.0–48.3 months) and 11.4 months (range: 5.0–44.0 months), respectively. Of the 18 IS treatment responders, 2 (11.1 %) patients with pre-existing grade 2 COPD still required IS (400 μg twice a day) 45.0 and 48.3 months after radiotherapy, respectively. For the remaining 16 responders (88.9 %), IS therapy was stopped after 7.7 months (range: 3.0–18.2 months). None of the patients treated with IS developed any specific IS-related side effects such as oral candidiasis.

**Conclusion:**

This single-centre experience shows that high-dose IS is an individual treatment option for radiation-induced pneumonitis grade II in patients with a good performance status.

## Introduction

Lung cancer is a leading cause of cancer deaths worldwide [21] and is frequently treated with irradiation. Radiation pneumonitis (RP) in lung cancer patients usually occurs within 1 to 3 months after radiotherapy [38]. The optimal dose of radiotherapy is often limited due to normal lung tissue constraints [22]; particularly, RP is one of the most significant dose-limiting factors in the radiation treatment of non-small cell lung cancer (NSCLC; [3, 23]).

The lung volume that is irradiated is of great importance. When smaller lung volumes are irradiated (e.g., in breast cancer), clinically relevant RP rates are relatively low (<3 %), and pneumonitis is often transient and clinically mild [19, 20]. The use of higher radiation doses and the irradiation of larger lung volumes in combination with chronic lung diseases results more likely in clinically relevant pneumonitis [11, 18]. In approximately 25–30 % of lung cancer patients, mild to severe RP can be observed following definitive radiotherapy with 60–70 Gray (Gy) [11, 13, 15].

The clinical symptoms of RP include dyspnea, non-productive cough, pleuritic chest pain, fever and, rarely, acute respiratory distress syndrome (ARDS; [5, 6, 27]). In addition to the clinical symptoms, lung function parameters such as vital capacity (VC), forced expiratory volume (FEV1) and diffusion-capacity (D_LCO_) might be helpful in quantifying the impact of RP [7].

In a prospective study on the prevention of RP in 57 lung cancer patients, the authors supported the continuous application of steroids during the course of and following radiotherapy for preventing RP when the use of inhalative beclomethasone was superior to oral prednisolone in terms of better local efficacy and decreased unwanted side effects [22].

The most recent S2 guideline from the German Society for Radiation Oncology (DEGRO) recommends oral steroids for the symptomatic therapy of clinically relevant RP (DEGRO S2 guideline, Version 1.2, February 2015). Compared to inhalative steroids (IS), oral steroids have more pronounced side effect profiles; hyperglycaemia, weight gain, insomnia, osteoporosis, myopathy and cognitive disorders have been associated with long-term oral steroid treatment [4, 32].

In the presented analysis, we retrospectively assessed the efficacy of inhalative steroids in lung cancer patients with symptomatic RP grade II. Furthermore, as a secondary objective we tried to ascertain patient- and treatment-related parameters of IS resistance and performed an overall survival (OS) analysis.

## Material and methods

### Patients’ parameters

Between 05/09 and 07/10, 24 (female, *n* = 8; male, *n* = 16) patients with lung cancer were treated at a single institution with definitive chemoradiation (CRT) to the primary tumour site and involved hilar/mediastinal lymph nodes and developed grade II symptomatic RP. The presence of RP was recorded according to the Common Toxicity Criteria version 4.0 (National Cancer Institute Common Terminology Criteria for Adverse Events [CTCAE]).

In four of the patients (16.6 %), chemotherapy was omitted due to comorbidities, and in two of the patients (8.3 %), sequential chemotherapy was administered. Histologic analysis revealed NSCLC (*n* = 19) and SCLC (*n* = 3). The cancer histology could not be determined in 2 of the patients because sampling was considered to be too dangerous due to anticoagulation. The UICC stage distribution was as follows: IB, one patient (4.2 %); IIB, three patients (12.5 %); IIIA, four patients (16.7 %); IIIB, five patients (20.1 %); IV, eight patients (33.3 %); and limited-stage SCLC, three patients (12.6 %). The mean patient age at the start of CRT was 66 years (range: 50–82 years), and the mean follow-up period after CRT was 18.1 months (range: 4–65 months). The patient and treatment characteristics are summarised in Table 1.

**Table 1:** Patients’ characteristics and statistical analysis. IS non-responders were defined as patients with no significant improvement of clinical symptoms to the inhalative therapy after 2 weeks

	Inhalative steroid non-responders	Inhalative steroid responders	*p*-value
	*n* = 6	*n* = 18	
Median age (years)	63.3	65.2	0.680
Range (years)	52–74	50–82	
Gender			0.651
Male	4	12	
Female	2	6	
UICC stage			
IB	0	1	0.762
IIB	1	2	0.696
IIIA	0	4	0.304
IIIB	2	4	0.662
IV	4	5	0.182
Histological type	0	6	
Squamous cell carcinoma	2	8	0.545
Adenocarcinoma	3	6	0.283
Smoking status			
Non-smoker	1	4	0.634
Former smoker	5	13	0.520
Current smoker	0	1	0.750
Pack years (average)	52	47	0.131
Pre-existing COPD	2	7	0.603
ECOG status before radiotherapy	0		0.590
0	3	10	
1	3	8	
Spirometry before radiotherapy			
FEV1 (average, l)	1.73	1.8	0.705
VC (average, l)	2.9	2.9	0.544
RAWtot (average, kPas/l)	2.28	3.0	0.630
pO2 (average, mmHg)	70	67	0.686
pCO2 (average, mmHg)	36.8	40.1	0.207
HbCO (average, %)	0.3	2.2	0.077
Hb (average, g/dl)	15.45	14.05	0.424
DLCO (average, mmol/min/kPa)	4.4	4.8	0.701
Median total dose (Gy)	59	63	0.537
Mean lung dose (Gy)	14.9	15.4	0.475
Range	8–20	3–23	
Chemotherapeutic regimen			
None	1	2	0.597
Concurrent	4	16	0.366
Sequential	1	0	0.730

### Radiotherapy

In all of the patients, radiotherapy was based on a planning CT scan (slice thickness 3 mm) and three-dimensional (3D) treatment planning. The gross tumour volume (GTV) of each tumour was identified by computed tomography, PET, and bronchoscopy. Mediastinal lymph nodes with short axis diameters ≥1.0 cm and pretreatment PET scans with standardised uptake values (SUV) >3 were included. The clinical target volume (CTV) was defined as the GTV plus a 0.5 cm margin to account for microscopic tumour extension. For the planning target volumes (PTV), the CTVs were enlarged to allow for organ motion and set-up variation, and they were expanded by at least 1 cm in all directions. Definitive image-guided hypo-fractionated stereotactic radiotherapy (hfSRT) was performed in three of the patients (12.5 %). In these patients, on-board cone-beam computed tomography was used to confirm the correct positioning. Set-up errors were corrected before each irradiation treatment. For hfSRT, the CTV included GTV with a safety margin of 2–3 mm, and the PTV was defined as the CTV with a lateral safety margin of 5 mm and a craniocaudal safety margin of 5–8 mm. One patient (4.2 %) received conventional radiotherapy followed by sequential chemotherapy.

In general, irradiation was administered using a conformal multifield technique with 6- to 10-MV photons that were delivered by a linac accelerator. Quantitative dose-volume analysis was performed using cumulative dose-volume histograms (DVH) to ensure that the mean lung dose (MLD) was below 15 Gy and that the mean V20 and V30 were below 27 % and 20 %; the median dose for CRT was 60.0 Gy (range: 40–70 Gy), with daily single doses of 2.0 Gy (*n* = 20; 83.3 %). The single doses for hfsRT (*n* = 4; 16.7 %) were 6.0 and 12.5 Gy, which summed to the total doses of 37.5 Gy and 56 Gy, respectively.

### Diagnosis and treatment of RP

RP was diagnosed based on the typical clinical symptoms such as new or increased dyspnea, non-productive cough, pleuritic chest pain and fever. In each patient, a computed tomography (CT) scan of the chest was performed within a week to radiographically verify the RP diagnosis. A second CT scan was performed six to eight weeks later, and after another six to eight weeks, a third CT was performed. Thereafter, a chest and abdominal CT was routinely performed every 3 months or in cases of clinical signs of progressive disease. Each patient had an oncological follow-up visit after each CT scan. All patients had RP grade II, because no patient presented with severe symptoms requiring oxygen therapy and / or hospitalization [28]. Patients were informed that IS therapy in symptomatic RP represents an individual treatment which deviates from current guidelines [33]. All patients gave their consent. 

RP treatment was initiated with the inhalative steroid budesonide, at a dose of 800 μg twice daily (Pulmicort® 800 μg 1-0-1). One patient (4.2 %) required a non-steroidal anti-inflammatory drug for pain relief due to pleuritic chest pain. Upon the demands of two patients (8.3 %), simultaneous therapy with long-lasting ß_2_-sympathomematic salmeterol (50 μg twice daily) was initiated. Within 2 weeks of IS therapy initiation, the patients had a follow-up visit to assess treatment efficacy. If a patient’s clinical symptoms were not significantly ameliorated, the IS treatment was stopped, and the patient was switched to oral prednisolone (0.5 mg/kg bodyweight, with an initial dose of at least 50 mg per day). The prednisolone dose was halved every five days until the maintenance dose of 6 mg per day was attained; this dose was continued for an additional 6 weeks. The patients who showed no significant improvement of clinical symptoms within 2 weeks of IS treatment initiation were classified as non-responders. Vice versa patients who reported on mild improvement of symptoms or stable clinical symptoms were also classified as non-responders.

With the support of CT, IS therapy in the responders was tapered off. Within 6–8 weeks of the initial chest CT that was performed to confirm the RP diagnosis, the second chest CT was performed. If no increase but rather a consolidation of ground-glass infiltrates was found, the budesonide dose was halved to 400 μg twice daily (Pulmicort® 400 μg 1-0-1). If a patient showed no clinical symptoms of RP within 6–8 weeks of switching to this dose and another chest CT confirmed decreasing ground-glass infiltrates, the IS therapy was stopped.

### Statistical analysis

The statistical analysis was performed using a commercially available software package (SPSS V.22.0 for Windows). The patients were dichotomised into IS responders and IS non-responders, and they were compared according to gender, age, histology, various lung function parameters and lung doses Chi-square tests were carried out to assess the various patient- and treatment-related parameters and patient response to inhalative steroids, and a OS analysis using Kaplan-Meier curves and log-rank test was performed.

## Results

Symptomatic RP manifested a median of 2.8 months after radiotherapy (range: 1–5 months). No oxygen requiring (grade III), life-threatening (grade IV) or lethal (grade V) pneumonitis cases were observed.

In each patient, a chest CT confirmed the clinical diagnosis by showing blurred interstitial markings with spotty and partially confluent infiltrates, which are typical radiographic signs of pneumonitis.

According to the severity of the patients’ clinical symptoms, all 24 patients had RP grade II, because no patient required oxygen, which is the defining criterion for RP grade III.

Eighteen patients (75.0 %) were classified as responders to IS treatment; they presented with significantly decreased clinical symptoms within two weeks of IS treatment initiation. In all of these patients (18/18), the second CT, which was performed 6–8 weeks after the clinical and radiographic diagnosis of RP, showed decreased ground-glass opacities. This was confirmed in the subsequent CT, performed 6–8 weeks after. IS was taken for a median of 8.2 months (range: 3.0 – 48.3 months). Of the IS treatment responders, 2 (11.1 %) patients with pre-existing COPD grade II still required IS treatment (400 μg twice daily) at 45.0 and 48.3 months after radiotherapy, respectively. For the remaining 16 responders (88.9 %), IS therapy was stopped after 7.7 months (range: 3.0–18.2 months).

Of the 24 patients, 6 (25 %) showed no significant improvements in clinical symptoms two weeks after IS therapy initiation; in these cases, treatment was switched to oral prednisolone. In the 2-week interval after initiation of IS therapy until response assessment, in no patient neither an aggravation of clinical symptoms nor oxygen requiring dyspnea (RP grade III) was observed.

Prednisolone was taken for a median of 11.4 months (range: 5.0–44.0 months). Two patients required oral steroids until their death 16 and 44 months after radiotherapy due to brain metastases and drug-induced toxic diffuse alveolitis by amiodarone, respectively.

None of the patients who were treated with IS developed any specific IS-related side effects such as oral candidiasis. In contrast, every patient who was treated with oral steroids experienced at least one of the following side effects: appetite increase with weight gain, sleep disturbances, mood changes, and lower leg oedema. A patient with known diabetes experienced increased blood sugar values during treatment with oral steroids and required a new diabetes treatment.

At time of analysis, seven patients (29.2 %) were alive and free of disease, and seventeen (70.8 %) were deceased. None of the patients had died from fatal pneumonitis. One of the patients (4.2 %) who did not respond to IS died from congestive heart failure 20 months after radiotherapy. Sixteen (66.7 %) patients died from cancer-related conditions.

**Fig. 1:**
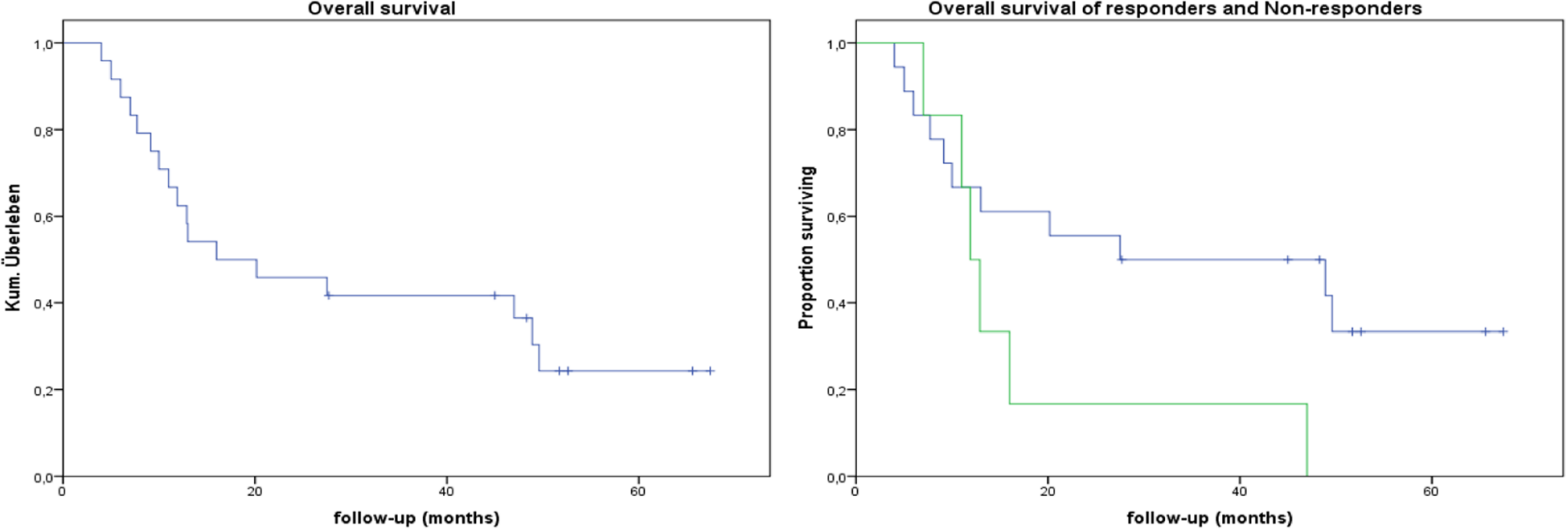
Overall survival of all patients (left), and differences (right) between responders (blue curve) and non-responders (green curve)

The patient and treatment parameters are summarised in Table 1. No statistically significant associations were found between the various patient- and treatment-related parameters and patient response to inhalative steroids. We observed a trend towards statistical significance (*p* = 0.077) with respect to HbCO level (0.3 % vs. 2.2 %). The median OS of the responders was 27.5 months compared with 11.9 months of the non-responders (*p* = 0.073; log-rank test). The Kaplan Meier curves and the survival chart are presented in Fig. 1 and Table 2, respectively.

**Table 2:** Results of the long-rank test showing in detail the cumulative proportion of survivors, number of deaths and patients at risk in each group at the different follow-up time points

Responder to IS	Follow-up (months)	Cumulative proportion of survivors	Number of cumulative deaths	Number of patients at risk
1	4	0.94	1	17
2	5	0.89	2	16
3	6	0.83	3	15
4	7.7	0.78	5	14
5	9.1	0.72	6	13
6	10.0	0.67	7	12
7	13.0	0.61	8	11
8	20.2	0.56	9	10
9	27.4	0.5	9	9
10	27.7	-	9	8
11	45.0	-	9	7
12	48.3	-	9	6
13	48.9	0.42	10	5
14	49.6	0.33	11	4
15	51.7	-	11	3
16	52.6	--	11	2
17	65.6	-	11	1
18	67.4	-	11	0
Non-Responder to IS				
1	7.0	0.83	1	5
2	11.0	0.67	2	4
3	11.9	0.5	3	3
4	12.9	0.33	4	2
5	16.0	0.17	5	1
6	47.0	0	6	0

## Discussion

RP is a fairly common subacute side effect of radiotherapy in lung cancer patients; it has a reported incidence of 10–30 %. This incidence range likely arises from different patient populations, the subjective scoring of RP and treatment-related factors [25].

The application of oral steroids is recommended for the treatment of symptomatic RP. However, the therapy is not causally but merely symptomatic; the progression of RF is unlikely to be influenced [9, 12, 31]. Several agents, such as TNF-alpha and TGF-beta inhibitors, have been tested as causal treatments for RP to interrupt the development of fibrosis; however, none of them have been been established in clinical practice [34].

Oral steroids suppress the symptoms of pneumonitis. In contrast to the oral application of steroids, the inhalative application of steroids has a lower risk of systemic side effects, such as weight gain, hyperglycaemia, sleep disturbances, mood changes and oedemas [4, 32]. In our study, at least one of these side effects was observed in each of the seven patients who did not respond to IS and required oral prednisolone. This is important because oral steroids must be taken for approximately two months to prevent the exacerbation of symptoms [24].

Pagel et al. [22] tried to reduce the incidence of RP with the prophylactic application of either oral or inhalative steroids; the authors compared the application of the systemic and inhalative approaches. Inhalative beclomethasone was found to be superior to oral prednisolone in terms of better local efficacy and decreased unwanted side effects. In line with these results, the application of inhalative steroids in the therapeutic setting seems to be consistent.

In the present study, IS were taken for a median of 8.2 months. Although this may appear to be a long time, the recommendation for taking oral steroids is at least two months to avoid symptom exacerbation ([24], DEGRO S2 guideline, Version 1.2, February 2015). We decided to slowly taper off IS treatment; the IS dose was reduced only when a chest CT scan 6–8 weeks after initiation of IS therapy confirmed the regression of the typical radiographic signs of RP. The efficacy of this strategy was confirmed by the fact that none of the responders experienced symptom exacerbation during the stepwise reduction of IS treatment.

Six patients (25 %) did not report on a significant improvement of clinical symptoms after two weeks of IS and were classified a non-responders. Their therapy was switched to oral prednisolone. No statistically significant differences were found in the various patient- and treatment-related parameters, including spirometric measures, before radiotherapy between the responders and non-responders. Several of the patient- and treatment-related parameters may have influenced the patient response to IS.

Important treatment-related parameters include the dose-volume parameters of radiotherapy [29] and application of chemotherapy [16]. To prevent the occurrence of RP, the irradiated volume should be as low as possible. There is considerable evidence that the risk of late lung toxicity is a function of dose-volume parameters including the mean lung dose (MLD) and certain volumes of the lung that receive different cumulative doses. As a general rule, the risk for symptomatic RP sharply increases with a MLD >20 Gy, V20 > 30 % and V30 > 20 %, respectively [29, 35, 37]. For our patients, the MLD was kept below 15 Gy, and the mean V20 and V30 were kept below 27 % and 20 %, respectively. Nevertheless, there is no general threshold dose for developing symptomatic RP [1].

In some studies, chronic obstructive pulmonary disease (COPD) was associated with radiation-induced lung toxicity [30], whereas other studies reported no statistically significant relationship [8, 36]. However, pre-existing COPD may have contributed to patient non-response to IS because clinical symptoms, including dyspnea, are also typical of COPD exacerbation; thus, an overlap between COPD and RP may have contributed to patient non-response to IS.

Older age and the application of concurrent chemotherapy seem to be risk factors for more severe RP after radiation [12, 16], while active smoking seems to have a protective effect [10, 26]. However, opinions vary regarding the effects of smoking on RP, and some studies have identified smoking as a risk factor for pneumonitis [26]. We found a trend towards lower HbCO levels in non-responders (0.3 % vs. 2.2 %), which potentially reflects patient smoking habits, although only 1 of the patients who responded well to the IS therapy was an active smoker. There might be a bias between the responder and non-responder groups because we only collected information on smoking status at the time of diagnosis. We did not know whether any of the patients started smoking after their diagnosis again or made false statements.

While smoking may be a protective factor against developing RP [9], smoking could also be a factor for better inhalative IS treatment response compared to quitting smoking or never smoking at all. This might be due to changes in the immune response of smokers compared to non-smokers [14].

None of the non-responders were alive at time of analysis. We are convinced that there was no relationship between non-response and poor survival because all of the patients showed good symptom relief after initiating oral corticosteroid therapy. Rather, 3/6 (50.0 %) patients initially presented with UICC stage IV lung cancer, and two (33.3 %) of the patients had UICC stage IIIB lung cancer, which are generally associated with very poor prognoses [2]. Furthermore, Inoue et al. [16] showed in a series of 256 lung cancer patients that mild RP had in contrast to severe RP no impact on the overall survival. This suggests that steroids act only as symptomatic therapy but do not prevent lung tissue damage.

The limitations of our study include its retrospective design and the relatively small sample size. The small sample size likely influenced the statistical analysis because we were unable to show significant differences between the IS treatment responders and non-responders. Therefore, it was not possible to identify treatment- and patient-related parameters associated with patient response or non-response to inhalative budesonide. Furthermore, therapy was controlled on the basis of the severity of clinical symptoms and regression of radiographic signs of RP on the CT scans. We do not know whether the introduction of regularly measured lung function parameters and blood gas analysis would have improved patient selection for IS therapy and the response rate to IS therapy, although blood gas analysis failed to show utility for diagnosis and treatment of RP in the largest published series with 385 patients by Sekine et al. [31]. The treatment of RP still depends on the experience of the clinician [16, 31]. Additionally, there are no data about the time interval, in which a response to IS can be expected. Therefore, we do not know if the interval from initiation of IS therapy until clinical response assessment was too long, although RP is known to responds better to steroids than drug-induced interstitial lung disease [17].

As even no aggravation of clinical symptoms in the group of non-responders to IS therapy was observed, and only the lack of significant improvement of the clinical symptoms led to the decision to switch inhalative budesonide to oral prednisolone, we believe that our approach in this selected cohort of patients was safe. Additionally, it is important to emphasize that no patient with an ECOG Score ≥2 was selected for IS therapy. Therefore, no general statements can be made about patients with poor performance status.

However, despite these limitations, we are convinced that our report makes useful contributions to the discussion on the individualised treatment of symptomatic RP grade II in lung cancer patients after radiotherapy.

## Conclusion

As an individual treatment in patients with a good performance status, symptomatic RP grade II in lung cancer patients after radiotherapy can be initially treated with inhalative steroids, leaving the application of oral steroids for non-responders. Requirements are close observation of the patient and considerable experience with RP of the clinician.
